# Using Speed and Accuracy and the Simon Effect to Explore the Output Form of Inhibition of Return

**DOI:** 10.3390/vision7010025

**Published:** 2023-03-20

**Authors:** Ralph S. Redden, Matthew D. Hilchey, Sinan Aslam, Jason Ivanoff, Raymond M. Klein

**Affiliations:** 1 Department of Psychology & Neuroscience, Dalhousie University, Halifax, NS B3H 4R2, Canada; 2 Department of Psychology, University of Alberta, Edmonton, AL T6G 2E9, Canada; 3 Rotman School of Management, University of Toronto, Toronto, ON M5S 3E6, Canada; 4 Department of Psychology, Saint Mary’s University, Halifax, NS B3H 3C3, Canada

**Keywords:** orienting, inhibition of return, Simon effect, cueing, information processing dynamics

## Abstract

Inhibition of return (IOR) refers to slower responses to targets presented at previously cued locations. Contrasting target discrimination performance over various eye movement conditions has shown the level of activation of the reflexive oculomotor system determines the nature of the effect. Notably, an inhibitory effect of a cue nearer to the input end of the processing continuum is observed when the reflexive oculomotor system is actively suppressed, and an inhibitory effect nearer the output end of the processing continuum is observed when the reflexive oculomotor system is actively engaged. Furthermore, these two forms of IOR interact differently with the Simon effect. Drift diffusion modeling has suggested that two parameters can theoretically account for the speed-accuracy tradeoff rendered by the output-based form of IOR: increased threshold and decreased trial noise. In Experiment 1, we demonstrate that the threshold parameter best accounts for the output-based form of IOR by measuring it with intermixed discrimination and localization targets. Experiment 2 employed the response-signal methodology and showed that the output-based form has no effect on the accrual of information about the target’s identity. These results converge with the response bias account for the output form of IOR.

## 1. Introduction

In spatial cueing paradigms, responses are usually slower to targets at previously cued relative to uncued locations at cue-target onset asynchronies between 0.2 and 3 s [1,2]. The finding is often referred to as inhibition of return (IOR; [3,4]) and it is thought to promote efficient visual search by discouraging re-inspection of previously processed stimulus locations [5,6].

Some studies show that the nature of IOR is different depending on whether eye movements are made. When eye movements are not made, slower responses are observed only when the target appears within the vicinity of the cue [7,8,9,10]. Because this effect depends on repeated stimulation of an input pathway, it is commonly described as attentional/perceptual [7] or, synonymously, as occurring nearer the input end of the information processing continuum [9]. When eye movements are made, responses toward the cued location are slower than responses away from it, even when responding to a centrally presented arrow [2,7,11]. Because this effect does not depend on repeated stimulation of an input pathway, it is commonly described as motoric/decisional [7] or, synonymously, as occurring nearer the output end of the information processing continuum [12].

**Figure 1:**
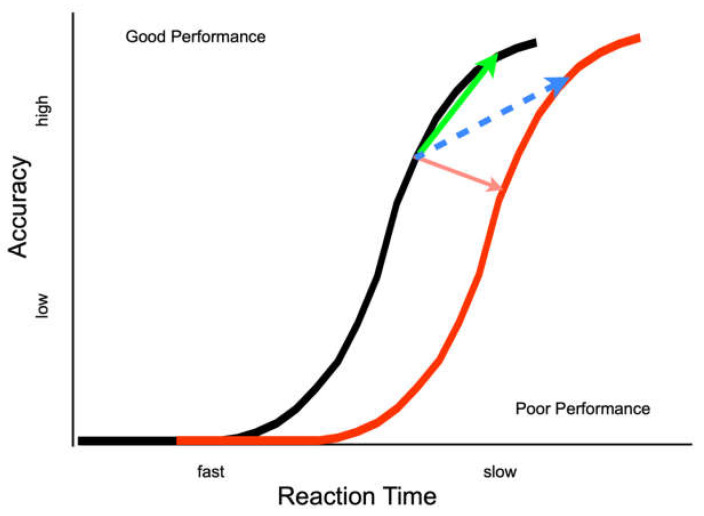
Theoretical depiction of the two theories of IOR. All functions represent typical functions relating the improvement in the accuracy of performance as response time increases when participants are responding to targets that appear and are neither masked nor removed. If IOR causes a genuine deterioration in performance this would result in a rightward shift of the function (as shown by the red arrow) or a change in slope of the function (not depicted in this figure). Evidence for these results has been shown in studies where eye movements were prohibited [14]—Exp 3A, [15]—Antisaccade condition [18]. Another possible pattern, where participants demonstrate slower but more accurate responding (also referred to as a speed-accuracy trade-off or criterion shift), has been shown when eye movements were made [14]—Exp 1B and 3B; [15]—Prosaccade condition] or when participants were instructed not to make them but eye position was not monitored [19] and the eye movement system may not have been effectively suppressed (as shown by green and blue arrows). In this, and each subsequent figure, the arrows indicate the direction of the effect of the cue (e.g., from uncued to cued).

Ivanoff, Klein and Lupianez [13] suggested two different effects of IOR (Figure 1). An inhibitory effect closer to the input end of the information processing continuum would be reflected by a rightward shift in the information accrual function for cued relative to uncued targets (i.e., a genuine decrease in information processing efficiency at the cued location). An inhibitory effect closer to the output end of the information processing continuum would be reflected by a response bias or criterion shift (i.e., a greater evidential threshold for responding). Recent investigative work has focused on experimentally testing when and whether to expect these kinds of effects of IOR.

For example, Chica, Taylor, Lupianez and Klein [14] administered a spatial cueing paradigm in which they manipulated, between subjects, whether a participant made an eye movement toward a spatially uninformative peripheral cue and back to fixation (pro-saccade condition) or not (no-saccade condition). Shortly thereafter, participants had to discriminate the color of a target with a keypress response. Two qualitatively different patterns were obtained, each corresponding to the theoretical constructs posited by Ivanoff, Klein and Lupianez [13]. In the no-saccade condition, responding was slower and less accurate to targets at the cued location, suggesting an effect on input processes. In the pro-saccade condition, responding was slower but also more accurate at the cued location, suggesting an effect on output processes.

Redden, Hilchey and Klein [15] extended Chica et al.’s findings [14] by replacing the no-saccade condition with an anti-saccade condition (i.e., an eye movement was to be made to the location opposite the cue). They did this to test the hypothesis from Klein and Hilchey [16]; see also [6] that the critical factor in determining the form of IOR is not whether eye movements are involved (i.e., [7]) but rather whether eye movements are permitted toward cues and targets in peripheral vision. Redden et al. replicated Chica et al.’s output-based IOR effect when eye movements were made to the cue. However, anti-saccades to the cue led to input-based IOR effects. According to Klein and Hilchey, this dissociation occurred because it was necessary to suppress the reflexive oculomotor machinery in order to make anti-saccades (but not in order to make pro-saccades). They theorized that it is whether this machinery is in a tonically suppressed or active state that determines whether input- or output-based forms of IOR will be observed (see also [9,17]).

Recently, drift diffusion modeling (e.g., [20]) has provided converging evidence that the input- and output-based forms of IOR arise from dissociable mechanisms. Diffusion models (e.g., [21,22]) assume a stochastic approach to evidence accumulation. There are a number of latent processes in the decision-making process (e.g., drift rate, thresholds, starting points), inherent in the model, which is estimated from observed performance measures (i.e., distribution of correct and incorrect RTs). Redden, MacInnes and Klein [23] applied diffusion modeling to the data from Redden, Hilchey and Klein [15] and found that the input-based form of IOR generated in the anti-saccade condition was best accounted for by a reduction in the drift rate parameter. The drift rate parameter represents the average slope at which information accrues toward the “Correct” response in a random walk model of a 2AFC, and thus a reduction of this slope would produce slower RTs (because the information is not accruing as fast) and less accurate responses overall (since reducing slope away from “Correct” necessitates it is more sloped toward “Incorrect”)—a pattern consistent with an effect on the quality of perceptual information processing. The output-based form of IOR generated in the pro-saccade condition was well fit by either (1) an increase in the response threshold parameter or (2) a reduction in the drift noise parameter. The response threshold parameter represents the distance between the start point and each of the two responses, or how far the random walk has to travel to reach one of the two response options. An increase in the threshold would result in slower RTs (because more information is needed) and more accurate responses overall (because more information results in greater accuracy). The drift noise parameter represents the magnitude of variability in the accrual of information as time progresses within a trial, representing the signal-to-noise ratio (SNR) within a trial. A reduction in drift noise would also result in slower RTs (because both signal and noise contribute to overall RT) and more accurate responses (because less noise is interfering with signal). Whereas an increased threshold account would be consistent with an output effect, it is ambiguous whether reducing drift noise would suit such a theoretical construct. The conceptualization of an output effect is explicit that there is a reluctance to respond as a consequence of the cue (change in threshold), not that the information at the cued location has been influenced (change in SNR). The main goal here is to resolve this ambiguity by determining experimentally which behavioral effect best characterizes output-based IOR (Experiment 1) and to help clarify the nature of this phenomenon (Experiment 2).

## 2. Experiment 1

In Experiment 1, we aimed to resolve this ambiguity by intermixing centrally presented arrow targets with the peripheral targets in Redden, Hilchey and Klein’s [15] pro-saccade condition. The arrows at fixation pointed to the left or right and required left and right keypress responses, respectively. The peripheral targets appeared to the left or right of fixation and their shapes were discriminated with left and right keypress responses. All targets were preceded by a transient, spatially uninformative cue on the left or right side of fixation that called for a pro-saccade. In this design, we expect that discrimination responses will be slower but more accurate to cued as compared to uncued targets in peripheral vision, which is diagnostic of output-based IOR [14,15]. More importantly, the design provides two additional diagnostics useful for resolving the aforementioned ambiguity.

The first diagnostic as to whether the response threshold or drift noise better accounts for output-based IOR concerns whether the arrow-elicited responses are affected by their compatibility with the cued location (e.g., left cue, left arrow response = compatible/cued; left cue, right arrow response = incompatible/uncued). If IOR results from an increase in the response threshold toward the cued location, then arrow responses compatible with the cue location should be slower than arrow responses incompatible with it. If IOR results from a decrease in drift noise at the cued location, then responses to these arrows should be unaffected by the cueing, since there is no spatial overlap between the cues and central arrow targets.

A second diagnostic is provided by the Simon effect [24], which refers to the observation that responses are faster and more accurate for effectors nearest the target. The Simon effect has long been thought to be the result of response conflict [25]. Ivanoff, Klein, and Lupiáñez [13] summarize the numerous ways in which various proposals for the mechanism underlying IOR might interact with the Simon effect. In a meta-analysis, they observed an interaction between IOR and the Simon effect such that the Simon was greater for targets at locations impacted by IOR (see also [26]). Wang, Fuentes, Vivas, and Chen [27] have also observed this interaction, noting that the neural activity in the precentral cortex (i.e., primary sensorimotor cortex) may be the source of the interaction between IOR and the Simon effect. As discussed earlier, the absence of eye monitoring (and not knowing whether the reflexive oculomotor system is in a continuously suppressed state) poses a problem for interpreting the locus (input, output, or both) of IOR. To put this in perspective, Redden, Hilchey and Klein [15]—Supplementary Materials, demonstrated that the input and output forms of IOR have opposite interactions with the Simon effect, with input- and output forms increasing and decreasing Simon effects, respectively (Evaluation of the Simon effect is a convenient but incidental consequence of response mappings [‘z’ and ‘/’ keys] on the same spatial axis as target locations [left and right]). An enhancement of the Simon effect is consistent with an increased tendency toward the prepotent response when target signal quality is reduced, as concluded by Hilchey et al. [26] who found evidence that IOR delayed the processing of the task-irrelevant spatial stimulus-response information activated automatically by the target’s location more so than it delayed the processing of the non-spatial stimulus-response information activated by the target’s task-relevant identity. In contrast, an attenuation of the Simon effect is precisely what would be expected if there were a reluctance to make responses compatible with the location of the cue, which would be consistent with an effect on response threshold, but not drift noise.

### 2.1. Method

#### 2.1.1. Participants

Twenty-four (one left-handed; five male) naive participants ranging in age from 17 to 24 (M = 19.8) participated in the study in a 60 min session. Participants were compensated at a rate of either 1.0-course credits or $12 per hour. All participants were recruited from the undergraduate subject pool at Dalhousie University. Experimental protocols were approved by the Dalhousie University Research Ethics Board (protocol code 2014-3396, 04/11/2014).

#### 2.1.2. Apparatus and Procedure

**Figure 2:**
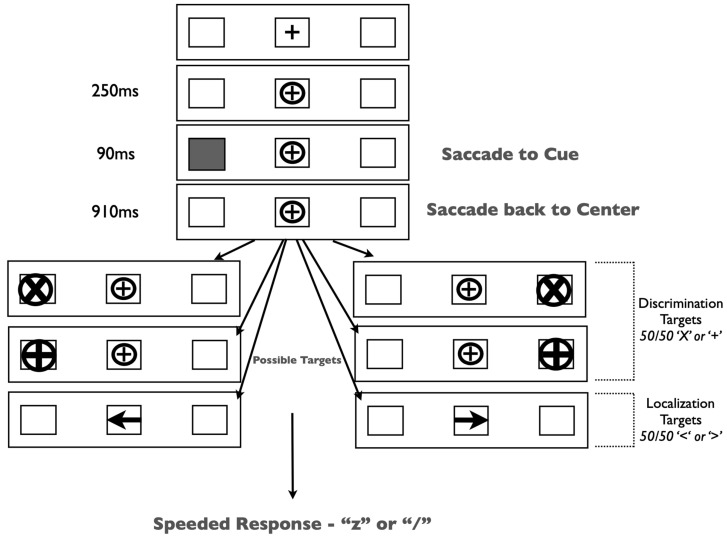
Methods figure depicting the time course of a trial. Duration of each subsequent event is depicted to the left of the image. Discrimination targets were counter-balanced for location (left or right placeholder) and identity (‘X’ or ‘+’). Localization targets were counter-balanced for direction (left or right pointing). The relative frequency of target type was manipulated between groups. This image is not to scale, so the relative size of features may be misrepresented.

The experiment was run in a dimly lit room on a 19” CRT monitor. Gaze position was monitored continuously by EyeLink II head-mounted eye tracking equipment. Our stimuli and procedure (Figure 2) were identical to those in Redden, Hilchey and Klein [15] except for a single change: participants were presented with one of two target types on each trial—peripheral x/+ discrimination or central left/right arrow targets.

Trials began with the presentation of three black outline placeholder boxes [1.5 × 1.5 degrees visual angle (DVA)] separated horizontally by 6.2 DVA on a grey background. The center box contained a black ‘+’ (0.5 DVA) as a fixation stimulus. Trials began with a drift correction that required the participant to fixate on the central stimulus and press the spacebar. If the participant was not fixating on the central stimulus, then a tone alerted them to refixate. Upon fixation, a circle subtending 0.9 DVA encircled the fixation stimulus and remained onscreen for the duration of the trial. Two hundred fifty milliseconds (ms) after the appearance of the circle, one of the lateral placeholder boxes was cued by filling in the empty space with grey. This stimulus lasted for 90 ms and did not predict the target location. Participants were required to generate a saccade to the cued placeholder box and back to the fixation stimulus. Trials on which inaccurate (>3.0 DVA from the target or center location) or early eye movements occurred (i.e., prior to cue onset) were terminated and recycled. After the successful eye movements, participants were instructed to maintain fixation for the duration of the trial. The target type was randomly selected on each trial. In discrimination trials, a target was presented in one of the lateral placeholder boxes (50% left, 50% right) 1000 ms after the onset of the cue. These targets were equally likely to be either an ‘X’ or a ‘+’ within a circle (1.3 DVA). Participants were required to identify the target by pressing either the ‘z’ or ‘/’ keys, respectively. Arrow targets (1.0 DVA) were presented in the central placeholder box pointing either left or right. These targets required a speeded response indicating the direction of the arrow (‘Z’ for left; ‘/’ for right). All targets remained on the screen until response. Participants completed 32 practice trials, followed by 320 experimental trials.

Experimental code, data, and analysis scripts for Experiments 1 and 2 can be found at https://osf.io/gz4ns/.

### 2.2. Results

Trials on which inaccurate eye movements occurred prior to target onset were excluded (0.9%). Based on visual inspection of the overall RT histograms, anticipatory target responses (<250 ms arrow: 0.1%; <300 ms discrimination: 0.2%) and slow target responses (>800 ms arrow: 3.5%; >1200 ms discrimination: 3.2%) were excluded from the analysis. After these exclusions, one participant was removed due to performance being close to chance in the discrimination task (Accuracy < 60%), leaving an N of 23 for analysis.

Generalized linear mixed-effects models were used (GLMER—lme4 R package [28]) to examine the trial-by-trial relationship between predictor variables—Cueing and Simon—and the outcome variables—Reaction Time (gaussian function) and Correct/Incorrect (binomial logistic link function). The model did not converge when each predictor was treated as both a fixed and random effect; however, removing Simon as a random effect afforded convergence. The interaction model was run first, followed by the main effect model, with AICs computed via the drop1 method in the {stats} package. Effect sizes for parameter estimates are reported as bootstrapped 95% confidence intervals, generated via confint.

#### 2.2.1. Peripheral Discrimination Task

**Figure 3:**
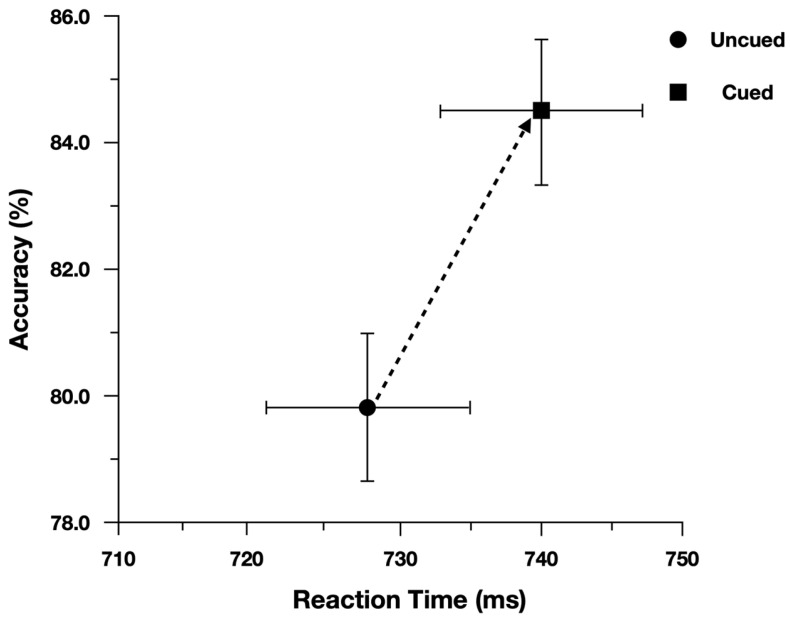
Overall performance for discrimination targets plotted in speed-accuracy space. Error bars represent Fisher’s least significant difference.

**Figure 4:**
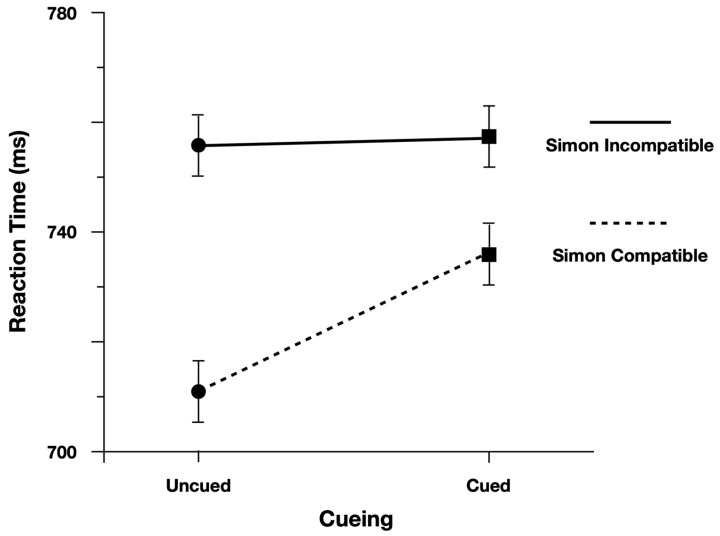
Reaction time to discrimination targets split by Simon Compatibility (solid line = Simon Compatible; dashed line = Simon Incompatible). Performance to cued and uncued targets is represented on the *x*-axis. Error bars represent Fisher’s least significant difference.

We performed the analysis on log(Correct RT), and reported effect estimates in log space. However, for ease of interpretation we plotted Correct RT. When examining Correct Reaction Time, participants were slower (13 ms) and more accurate (4.5%) when responding to cued peripheral discrimination targets (Figure 3). Moreover, as predicted, the Simon effect was reduced for Cued targets (24 ms) relative to Uncued targets (47 ms; Figure 4). There was evidence to support the two-way interaction, Cueing x Simon, b = 0.029, CI95% = [−0.005, 0.060], as the model performed worse with the interaction term dropped (AIC = −971) than when the term was included (AIC = −973).

To evaluate the main effects, we contrasted the model with both main effect terms included (AIC = −971) with models where each term was dropped. The model performed worse (^Δ^AIC = +1) when dropping the main effect of Cueing (Cued = 740 ms, Uncued = 728 ms), b = −0.018, CI95% = [−0.037, 0.001]. The model also performed worse (^Δ^AIC = +36) when dropping the main effect of Simon (Simon Compatible = 723 ms, Simon Incompatible = 759 ms), b = 0.047, CI95% = [0.033, 0.062].

When examining Proportion Correct, there was no evidence to support the two-way interaction, Cueing × Simon, b = −0.168, CI95% = [−0.567, 0.194], as the model performed better with the interaction term dropped (AIC = 3013) than when the term was included (AIC = 3014).

To evaluate the main effects, we contrasted the model with both main effect terms included (AIC = 3013) with models where each term was dropped. The model performed worse (^Δ^AIC = +11) when dropping the main effect of Cueing (Cued = 84.5%, Uncued = 79.7%), b = 0.422, CI95% = [0.182, 0.647]. The model also performed worse (^Δ^AIC = +92) when dropping the main effect of Simon (Simon Compatible = 87.9%, Simon Incompatible = 75.9%), b = 0.911, CI95% = [0.686, 1.137].

#### 2.2.2. Central Arrow Task

**Figure 5:**
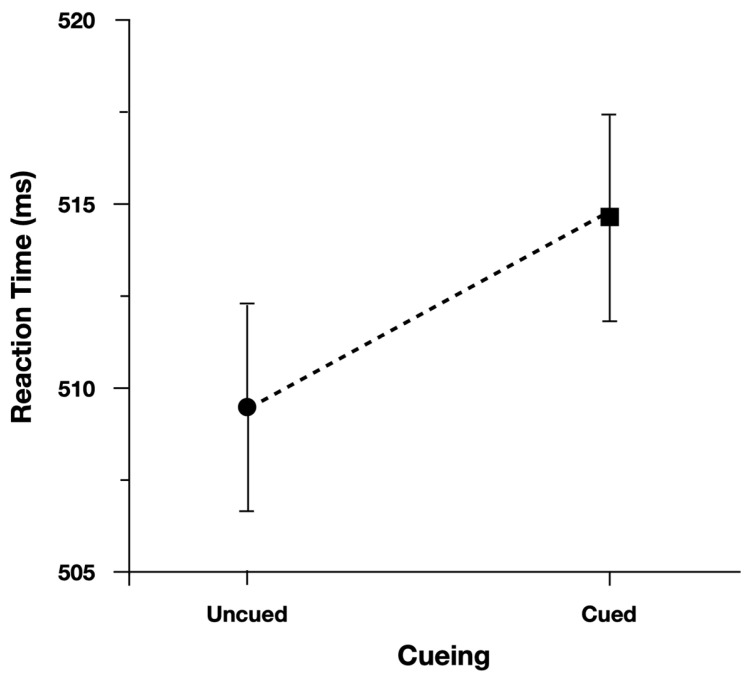
Reaction time to arrow targets. Performance to cued and uncued targets is represented on the *x*-axis. Error bars represent Fisher’s least significant difference.

Accuracy was not analyzed due to so few errors recorded to these targets (Cued = 98.2%, Uncued = 98.4%). Participants were slower (5 ms) to respond to cued central arrow targets (Figure 5). There was evidence to support the main effect of Cueing, b = −0.012, CI95% = [−0.028, 0.000], as the model performed worse with the effect term dropped (AIC = −2359) than when the term was included (AIC = −2360).

### 2.3. Discussion

This experiment yields three basic findings with respect to the nature of IOR caused by a pro-saccade: (1) Participants showed slower and more accurate responses for cued discrimination targets, consistent with an output-based form of IOR that could be generated by an increase in either response threshold or drift rate noise. (2) Arrow responses in the direction compatible with the cue were slower than arrow responses in the direction incompatible with the cue, and (3) the Simon effect was reduced at cued locations. The latter two effects are unambiguously consistent with an effect of IOR on the response threshold.

The main point here is that we have provided empirical support for one of the two tenable mechanisms underlying the output form of IOR, as proposed by a computational model for the two forms of IOR [23]. The findings do not rule out possible effects on the drift noise parameter, but they do ensure effects on the response threshold parameter.

As a secondary point, it is worth noting that while meta-analysis of the literature reveals that the Simon effect is enhanced by IOR [13], it can also clearly be reduced by IOR, depending on the kind of IOR that is generated [15]. The result of the meta-analysis likely reflects an amalgam of different kinds of IOR from literature that, on balance, just happens to succeed more often at generating input-based forms.

## 3. Experiment 2

Experiment 1 provides converging evidence that output-based forms of IOR can affect response thresholds but there is still ambiguity about the exact nature of the effect. Namely, when the inhibition from the cue expresses itself as a speed-accuracy tradeoff (SAT), it is not possible to tell unambiguously whether the effect is a shift along one or to a different accrual function (see Figure 1—blue and green arrows). No such ambiguity exists when the inhibition from the cue is not expressed as a speed-accuracy tradeoff (SAT, see Figure 1—red arrow; e.g., [14,15,18,29]). SATs can be due to a criterion shift alone, with slower but more accurate responses to cued as compared to uncued targets on a single accrual function (Figure 1—green arrow), or due to a criterion shift plus a change in performance, with slower but more accurate responses to cued as compared to uncued targets on separate accrual functions (Figure 1—blue arrow).

The goal of Experiment 2 is to further clarify, the nature of the SAT elicited in Experiment 1, Chica et al. [14] and Redden, Hilchey and Klein [15] by way of the response signal methodology [26,29,30,31]. The response signal methodology has long been used to measure the ability of the observer to identify target features under varying time constraints. It generates functions in SAT space that represent accuracy as a function of response (or processing) time.

If the effect of IOR generated by an eye movement to a peripheral cue is best represented by an increase in the threshold parameter, ergo it is operating purely on the output stages of information processing, then a performance from cued and uncued targets will belong to a single accrual function (representing the same accrual of information with shifts along the function as a result of IOR—Figure 1 green arrow). In contrast, if IOR in this paradigm generates a shift to a less efficient function, then our proposal would be wrong and we would need to revise our thinking. Moreover, if the response-signal methodology allows for complete control over the speed of responding, no performance difference will be observed across cueing conditions. If there is some concomitant inhibitory effect on inputs, then the accrual function for cued (inhibited) targets will be shifted to the right (Figure 1—dashed blue arrow).

### 3.1. Method

#### 3.1.1. Participants

Eleven naive participants ranging in age from 19 to 32 participated in the study over five 60 min sessions, one of whom was excluded for an inordinately high rate of target fixations (70% of trials; all others < 18%). Participants were compensated at a rate of $12 per session. All participants were recruited from the undergraduate subject pool at Dalhousie University. Experimental protocols were approved by the Dalhousie University Research Ethics Board.

#### 3.1.2. Apparatus and Procedure

**Figure 6:**
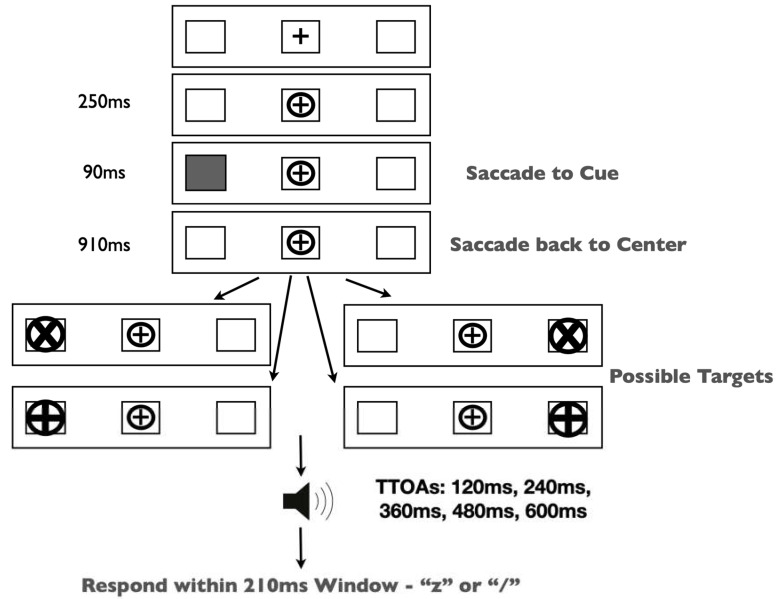
Methods figure depictfing the time course of a trial. Duration of each subsequent event is depicted to the left of the image. Participants were required to execute their response within the response window indicated by a tone presented at a single TTOA in a given block. This image is not to scale, so the relative size of features may be misrepresented.

Our stimuli and procedure (Figure 6) were identical to those in Experiment 1 except for two changes: the response window method (described below) was added (i.e., responses were constrained to a predetermined experimental criterion), and participants were only presented with peripheral x/+ discrimination targets (i.e., arrow targets were removed).

Keypress responses were to be enacted after the onset of a tone. The target-tone onset asynchronies (TTOA) were 120, 240, 360, 480 or 600 ms and the response window was 210 ms. The target remained present until a response was made or until the response window had closed. Feedback was given onscreen when anticipatory (“Too Early!”), late responses (“Miss!”) or untoward eye movements (“Inaccurate eye movement detected”) were made. TTOA was blocked within sessions and the order within a session was random. In each session, each participant completed five blocks of 80 trials, one for each TTOA, for a total of 400 trials per participant, per session. Across the five sessions, there were thus 2000 trials (1000 cued and 1000 uncued) per participant. We are aware of four published experiments that used the response-signal method to explore the effect of IOR upon the accumulation of target-related information, with targets that remained visible until the response: Ivanoff and Klein ([29], Experiment 1: N = 10; #Trials/TTOA = 400 and Experiment 2: N = 13; #Trials/TTOA = 400); Zhao et al. ([31], Experiment 2: N = 10; #Trials/TTOA = 224); Hilchey et al. ([26], Experiment 3: N = 10; #Trials/TTOA = 400). All four of these found significant IOR effects.

### 3.2. Results

Session One was considered practice and was excluded from the analysis. Trials with inaccurate eye movements prior to target onset were excluded (7.1%). Analyses were performed on trials for which a response was recorded within the response window, resulting in the exclusion of 19.2% of trials. A paired samples t-test was conducted on the number of responses falling outside the response window as a function of Cueing. These tests showed that there was no influence of cueing on the average frequency of early (Cued = 53, Uncued = 49; t(9) = 1.25, *p* = 0.24) or late (Cued = 82, Uncued = 83; t(9) = −0.33, *p* = 0.77) responses. Additionally, the inclusion of these trials in subsequent models changes no statistical patterns or conclusions. Trials for which an eye movement was recorded during target presentation were excluded (8.1%).

Generalized linear mixed-effects models were used (GLMER—lme4 R package [28]) to examine the trial-by-trial relationship between predictor variables—Processing Time (Processing Time = Time from Tone to Response + TTOA), and Cueing—and the outcome variable—Correct/Incorrect (binomial logistic link function). The model did not converge when each predictor was treated as both a fixed and random effect, however removing Processing Time as a random effect afforded convergence. The interaction model was run first, followed by the main effect model, with AICs computed via the drop1 method in the {stats} package. Effect sizes for parameter estimates are reported as bootstrapped 95% confidence intervals, generated via confint.

**Figure 7:**
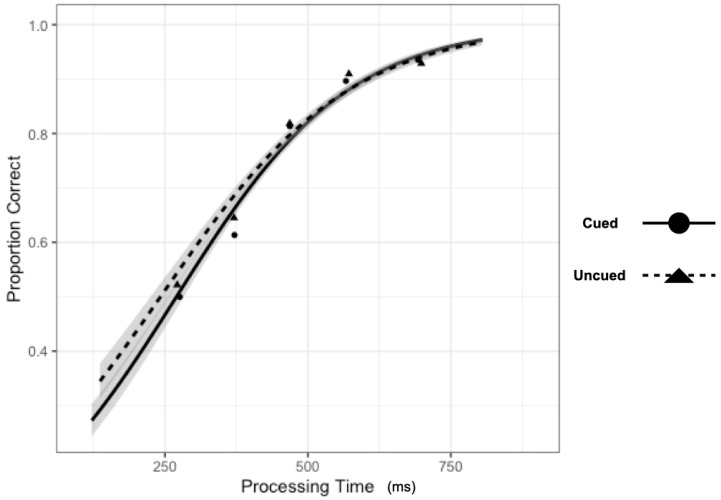
Information processing functions for cued (solid; circles) and uncued (dashed; triangles) performance. Processing time was calculated as the sum of the mean tone RT within a TTOA plus the TTOA. Grey shaded area reflects the 95% confidence interval for the fit of each cueing condition. Points represent the mean Processing Time and Proportion Correct for each Cueing condition for each of the five TTOAs.

There was little evidence to support the two-way interaction (Figure 7), Processing Time x Cueing, b = 0.0007, CI95% = [0.0000, 0.0013], as the model performed only slightly worse with the interaction term dropped (AIC = 12,773) than when the term was included (AIC = 12,771).

To evaluate the main effects, we contrasted the model with both main effect terms included (AIC = 12,773) with models where each term was dropped. The model performed substantially worse (^Δ^AIC = +1750) when dropping the main effect of Processing Time, b = 0.0064, CI95% = [0.0060, 0.0067]. Model performance was only slightly worse (^Δ^AIC = +1) when dropping the main effect of Cueing, b = 0.084, CI95% = [−0.0063, 0.164].

### 3.3. Discussion

These results suggest that input processing at cued and uncued locations is quite similar, with the proportion of correct discrimination responses being accounted for by a single information accrual function. This result would be expected if output-IOR were expressed purely as a criterion shift or post-perceptual effect. Further, since the same overt response was required at the same time to the same cue as in Experiment 1, the lack of a statistical cueing effect cannot be taken easily to suggest that there was no inhibitory consequence of the cue (i.e., IOR). That is, without the response signal methodology, these procedures robustly reveal that responses are slower when the target location is cued as compared to uncued. This pattern is qualitatively different from those reported by Ivanoff and Klein [29]. Eye movements to the cue were discouraged in their experiments, and they found IOR shifted the SAT function rightward (see [29]—Figure 5, see also [31]), consistent with the idea that IOR delayed the accrual of non-spatial (but task-relevant) information accrual (represented theoretically in the shift from black to red functions in the present Figure 1).

## 4. General Discussion

We have shown that when combining multiple measures of the output form of IOR in a single task (vis SAT pattern, arrow targets, relationship with the Simon effect), the effect is robust across the type of diagnostic. We observed inhibited performance for cued peripheral onset targets consistent with an SAT, inhibited performance for centrally presented arrows in the direction compatible with the cued location, and the attenuation of the Simon effect for cued targets. Each of these effects is consistent with the patterns predicted for a cue-elicited inhibitory mechanism that reduces the propensity to make responses in the cued direction.

We have utilized the response-signal methodology as a method for investigating the time course of information processing dynamics in the aftermath of inhibitory cueing effects. This method has allowed us to determine how full information processing functions in a 2-AFC task are affected by IOR when generated by overt, prosaccadic orienting, and contrast these findings with prior studies utilizing this methodology to evaluate the effect of the input form of IOR. This reinforces the theory that the output form of IOR is not affecting the quality of information accrued at the cued location and converges with that theory’s proposal that the output form is essentially a response bias.

We have assumed that the absence of a cueing effect in the SAT functions generated in E2 is because we generated the output form of IOR, which manifests as a speed-accuracy tradeoff, in combination with such a tight control over response times by the response signal methodology that not even an RT delay was generated. The skeptical reader could simply assume instead that, for some reason, IOR was not generated at all in E2. We cannot rule out this possibility, however, we know from numerous studies [7,11,14,15,17,32,33] that IOR is generated following pro-saccades. Moreover, we know from Ivanoff and Klein [29], Hilchey et al. [26], and Zhao et al. [31] that IOR can be generated when the response signal methodology is used. Therefore, we are interpreting the superimposed functions from E2 as support for the SAT characterization of the output form of IOR (green line in Figure 1), but we recognize that not all readers will be convinced (leaving open the possibility of the blue line in Figure 1).

When the present results are combined with the findings of the literature, it becomes clear that at least two qualitatively different mechanisms underlie IOR. Through the lens of drift-diffusion modeling, the two forms of IOR can definitely be captured by differences in the drift rate (input form) and response threshold (output form) parameters. The parameter(s) that will best capture the nature of IOR depends critically on the activation state of the oculomotor machinery for reflexive eye movements.

These findings provide converging evidence for the theory that the critical factor determining the type of IOR observed is the activation state of the reflexive oculomotor system. Furthermore, both forms of IOR show behavioral effects that would accomplish the novelty-seeking function attributed in the seminal papers by Posner and Cohen [1] and Posner et al. [2]—however, by altogether different mechanisms. The input form does so by decreasing the salience of recently attended inputs, whereas the output form does so by biasing orienting behaviors against previously attended locations.

## Data Availability

Experimental code, data, and analysis scripts for Experiments 1 and 2 can be found at https://osf.io/gz4ns/.
